# Evaluation of the SLOMYCO Sensititre^®^ panel for testing the antimicrobial susceptibility of *Mycobacterium marinum* isolates

**DOI:** 10.1186/s12941-016-0145-1

**Published:** 2016-05-06

**Authors:** Marion Chazel, Hélène Marchandin, Nicolas Keck, Dominique Terru, Christian Carrière, Michael Ponsoda, Véronique Jacomo, Gilles Panteix, Nicolas Bouzinbi, Anne-Laure Bañuls, Marc Choisy, Jérôme Solassol, Alexandra Aubry, Sylvain Godreuil

**Affiliations:** Département de Bactériologie-Virologie, CHRU Montpellier, Montpellier, France; UMR 5119 ECOSYM, Equipe Pathogènes et Environnements, Laboratoire de Bactériologie, U.F.R. des Sciences Pharmaceutiques et Biologiques, Montpellier, France; Laboratoire Départemental Vétérinaire de l’Hérault, Montpellier, France; Laboratoire Biomnis, Lyon, France; Department of Bacteriology-Virology, INSERM U1058 “Pathogenesis and Control of Chronic Infections”, Université Montpellier-EFS, CHU Arnaud de Villeneuve 371 avenue du Doyen Gaston Giraud, 34295 Montpellier, France; Université Montpellier, Montpellier, France; MIVEGEC (Laboratoire Maladies Infectieuses et Vecteurs, Ecologie, Génétique, Evolution et Contrôle), UMR CNRS 5290/IRD 224, Université de Montpellier, Montpellier, France; Department of Biopathology, CHRU Montpellier, Montpellier, France; Department of Clinical Oncoproteomic, Montpellier Cancer Institute, Montpellier, France; Centre National de Référence des Mycobactéries et de la Résistance aux Antituberculeux, Paris, France; Laboratoire de Bactériologie-Hygiène Paris, AP-HP, Hôpital Pitié-Salpêtrière, 75013 Paris, France; Centre for Immunology and Microbial Infections, team 13, Sorbonne Universités, UPMC Univ Paris 06, U1135, 75013 Paris, France; INSERM, U1135, Paris, France

**Keywords:** *Mycobacterium marinum*, SLOMYCO Sensititre^®^ panel, Agar dilution method, Antimicrobial susceptibility testing, Human and fish isolates

## Abstract

**Background:**

The agar dilution method is currently considered as the reference method for *Mycobacterium marinum* drug susceptibility testing (DST). As it is time-consuming, alternative methods, such as the E-test, were evaluated for *M.marinum* DST, but without success. The SLOMYCO Sensititre^®^ panel, recently commercialized by TREK Diagnostic Systems (Cleveland, OH), can be used for DST in slow-growing mycobacteria and for antimicrobial agents recommended by the Clinical and Laboratory Standards Institute (CLSI) for *M.marinum* DST. The main goal of this work was to evaluate the SLOMYCO Sensititre^®^ panel method for DST in *M.marinum* isolates from human patients and fish relative to the reference agar dilution method.

**Methods/Results:**

The reproducibility of the minimum inhibitory concentration (MIC) determination (±1 log_2_ dilution) was very good for both the agar dilution method and SLOMYCO Sensititre^®^ panel (>90 % agreement). The percentage essential agreement between methods varied, depending on the drug: between 97 and 75 % for ciprofloxacin, moxifloxacin, linezolid, isoniazid, clarithromycin, amikacin, rifabutin and rifampin, 74 % for trimethoprim, 72 % for doxycycline, 70 % for sulfamethoxazole, 59 % for streptomycin, 33 % for ethambutol and only 2.2 % for ethionamide. When the agar dilution and SLOMYCO Sensititre^®^ panel results were converted into interpretive criteria, the category agreement was 100 % for amikacin, ciprofloxacin, clarithromycin, moxifloxacin, rifabutin, sulfamethoxazole and trimethoprim, 98 % for ethambutol and 96 % for rifampin and no agreement for doxycycline.

**Conclusions:**

The SLOMYCO Sensititre^®^ panel method could provide a potential alternative to the reference agar dilution method, when DST in *M.marinum* is required, except for doxycycline.

## Background

*Mycobacterium marinum*, a slow-growing nontuberculous photochromogenic mycobacteria, is an ubiquitous waterborne organism [9, 10, 17] that causes diseases in many fish species from cold or warm, fresh or salted water, and also in many other aquatic animals, such as amphibians, mammals and oysters [9, 10, 17]. In humans, *M. marinum* infection is commonly limited to the skin, but it can spread to deeper structures, resulting in tenosynovitis, arthritis and osteomyelitis [1, 8, 13] and, rarely, in disseminated infection in immunocompromised patients [13].

*Mycobacterium marinum* is naturally multi-drug resistant and there is no standardized antimicrobial treatment for *M.marinum* infections [7]. As the wild-type susceptibility pattern of *M. marinum* is well known [2] and acquired resistance has not been described so far, antimicrobial susceptibility testing is not recommended except in the case of treatment failure and relapse [16].

The Clinical and Laboratory Standards Institute (CLSI) recommends microdilution for *M. marinum* drug susceptibility testing (DST) [16], but the agar dilution method is currently considered to be the reference method [2, 4, 19]. As this method is time-consuming, alternative methods, such as the E-test, were evaluated, but showed poor agreement with the reference method and therefore are not suitable for DST in *M.marinum* [2, 11, 19]. Recently, the SLOMYCO Sensititre^®^ panel was commercialized by TREK Diagnostic Systems (Cleveland, OH) [3]. This is a standard-order broth microdilution panel that can be used to evaluate the susceptibility of slow growing mycobacteria to 14 antimicrobial agents, including those recommended by CLSI for *M.marinum* DST [3].

The main goal of this work was to evaluate the SLOMYCO Sensititre^®^ panel method for DST in *M.marinum* strains from humans and fish relative to the reference agar dilution method.

## Methods

### Bacterial strains and growth conditions

The origin and other information concerning the tested *M. marinum* isolates are described in Broutin et al. [5]. The 35 *M. marinum* isolates from human patients and nine from fish were collected in France between 1995 and 2007. None was from patients who experienced treatment failure or relapse. *M. marinum* identification was performed using GenoType Mycobacterium AS/CM, a commercial multiplex line-probe assay (Hain Lifescience GmbH, Nehren, Germany). Isolates were stored at −80 °C in Middlebrook 7H9 broth (DIFCO, Detroit, MI, USA) containing 5 % OADC (DIFCO, Detroit, MI, USA) until determination of the Minimum inhibitory concentrations (MICs). Mycobacteria were then cultured in Löwenstein-Jensen (LJ) slants (bioMérieux, Marcy l’Etoile, France) and in Middlebrook 7H10 agar (DIFCO, Detroit, MI, USA). The *M. marinum* ATCC 927 strain (from fish) and the *M. marinum* ATCC BAA-535/M strain isolated from an infected patient were used as controls for MIC determination.

### Antimicrobial agents

Amikacin, ciprofloxacin, clarithromycin, doxycycline, ethambutol, ethionamide, isoniazid, rifampin, rifabutin, streptomycin and trimethoprim (Sigma-Aldrich, Lyon, France), linezolid (Pfizer, France) and moxifloxacin (Bayer, Wuppertal, Germany) were tested in this study. Stock solutions of each drug were prepared using the appropriate solvent and were filter-sterilized before storage at −80 °C. To prior testing, each drug was freshly diluted in sterile deionized water. The concentration ranges of the tested antimicrobial agents are indicated in Table 1.

**Table 1:** MIC (μg/mL) of the 14 antibiotics tested in 46 *M. marinum* isolates (35 clinical, 9 fish and 2 references strains), determined by using the agar dilution method

Antimicrobial agent [breakpoints (µg/mL)]^a^	MIC_50_			MIC_90_			Range
	Total (n = 46)	Human (n = 35)	Fish (n = 9)	Total (n = 46)	Human (n = 35)	Fish (n = 9)	
Amikacin (>32)	2	2	2	4	4	2	2–4
Ciprofloxacin (>2)	2	2	2	2	2	2	2
Clarithromycin (>16)	2	2	2	2	2	2	1–2
Doxycycline (>4)	4	4	4	4	4	4	1–4
Ethambutol (>4)	2	2	2	4	4	2	1–8
Ethionamide (NA)	5	5	5	10	10	10	2.5–10
Isoniazid (NA)	8	8	8	8	8	8	2–8
Linezolid (NA)	1	1	1	2	2	2	0.5–4
Moxifloxacin (>2)	1	1	1	1	1	1	0.12–1
Rifabutin (>2)	0.12	0.12	0.12	0.12	0.12	0.12	0.12
Rifampin (>1)	0.5	0.5	0.5	1	1	1	0.5–2
Streptomycin (NA)	16	16	16	16	16	16	4–32
Sulfamethoxazole (>38)	9.5	9.5	9.5	19	19	19	4.75–19
Trimethoprim (>2)	0.5	0.5	0.5	1	1	1	0.25–1

^a^Concentration range of the tested drugs by using the agar dilution method (ADM) and the SLOMYCO Sensititre^®^ panel method (SSPM): amikacin (0.25–32 mg/L, ADM; 1–64 µg/mL, SSPM); ciprofloxacin (0.12–16 µg/mL, ADM, SSPM); clarithromycin (0.06–32 µg/mL, ADM, SSPM); doxycycline (0.12–16 µg/mL, ADM, SSPM); ethambutol (0.5–16 µg/mL, ADM, SSPM); ethionamide (0.3–20 µg/mL, ADM, SSPM); isoniazid (0.25–16 µg/mL, ADM; 0.25–8 µg/mL, SSPM); linezolid (0.12–32 µg/mL, ADM; SSPM, 1–64 µg/mL); moxifloxacin (0.06–8 µg/mL, ADM; 0.12–8 µg/mL, SSPM); rifabutin (0.06–16 µg/mL, ADM; 0.25–8 mg/L, SSPM); rifampin (0.12–8 mg/L, ADM; 0.25–8 µg/mL, SSPM); streptomycin (0.5–32 µg/mL, ADM; 0.5–64 µg/mL, SSPM); sulfamethoxazole (2.38–152 µg/mL, ADM, SSPM); trimethoprim (0.12–8 µg/mL, ADM, SSPM); *NA* not available

### MIC determination by using the agar dilution method

The agar dilution method was performed on Müller-Hinton agar (Becton–Dickinson, France) supplemented with 5 % OADC according to CLSI [16]. Twofold dilutions of the antibiotics to be tested were added to obtain the required final concentrations. Confluent colonies of the different bacteria in LJ slants were swept with a loop and emulsified in sterile water and the concentration was adjusted to the McFarland n. 1 standard turbidity. A 1/100 dilution of each suspension adjusted to the McFarland n. 1 standard was inoculated using a Steers replicator to deliver approximately 10^4^ colony-forming units (CFU) per spot. Plates were incubated at 30 °C [16]. The MICs (i.e., the lowest concentration of antibiotic resulting in complete inhibition of growth) of the tested antibiotics were determined after 7 and 14 days of growth of the different *M. marinum* isolates/controls.

### MIC determination using the SLOMYCO Sensititre^®^ panel (broth micro-dilution method)

Inocula for the SLOMYCO Sensititre^®^ panel were prepared according to the CLSI and the manufacturer’s instructions [16]. SLOMYCO plates were incubated in a non-CO_2_ incubator at 30 °C until the controls showed sufficient growth (7–14 days). The MICs were determined visually using an inverted mirror and read as the lowest concentration of the antibiotic showing 100 % growth inhibition.

### Analysis of the results

The reproducibility of both methods was evaluated by performing two independent tests for each method and for each of the 44 isolates (a total of 176 tests) and five independent tests for the *M. marinum* ATCC 927 and ATCC BAA-535/M strains (a total of 30 tests). Each test result was independently interpreted by two blinded readers. The reproducibility value was defined as the percentage of strains with the same MIC value ±1 log_2_ dilution at each test. The essential agreement between the agar dilution and SLOMYCO Sensititre^®^ panel results was expressed as the percentage of isolates that showed the same MIC value ±1 log_2_ dilution with the two methods. Category agreement was evaluated using the breakpoints for determining the susceptibility and resistance categories recommended by CLSI [16]. For this study only very major errors (i.e., an isolate resistant [R] by the reference method, but susceptible [S] by the tested method) and major errors (S by the reference method and R by the test method) were considered. Minor errors (intermediate [I] by one method and S or R by the other method) were not considered because the CLSI breakpoints allow only two characterization categories (R or S) [16].

## Results

The reproducibility of the results obtained with the agar dilution method (reference method) was very good (Table 2) for all antibiotics. The MICs of the 14 antimicrobial agents determined by using the agar dilution method were distributed in a narrow range (Table 1). Comparison of the MICs for the *M.marinum* isolates from infected humans and fish did not reveal any difference.

**Table 2:** Reproducibility of the results (i.e., MIC, expressed in µg/mL, of the tested antibiotics in the 46 *M. marinum* isolates) obtained with the agar dilution method

Antimicrobial agents	No. of results within log_2_ concentration difference of							% agreement (confidence interval)
	>−2	−2	−1	0	1	2	>2	
Amikacin	0	0	0	45	1	0	0	100 (89–100)
Ciprofloxacin	0	0	4	33	7	2	0	96 (84.3–99.3)
Clarithromycin	0	0	10	23	13	0	0	100 (88–99)
Doxycycline	0	0	6	38	1	1	0	98 (87–100)
Ethambutol	0	0	2	43	0	1	0	98 (87–100)
Ethionamide	0	0	7	29	10	0	0	100 (90.4–100)
Isoniazid	0	0	5	21	20	0	0	100 (86–99.3)
Linezolid	0	1	12	20	12	1	0	96 (87–100)
Moxifloxacin	0	0	10	33	3	0	0	100 (90.4–100)
Rifabutin	0	1	3	28	14	0	0	98 (87–100)
Rifampin	0	0	12	30	4	0	0	100 (90.4–100)
Streptomycin	0	1	5	29	11	0	0	98 (87–100)
Sulfamethoxazole	0	0	10	25	11	0	0	100 (90.4–100)
Trimethoprim	0	0	10	26	10	0	0	100 (90.4–100)

The MICs for the two reference *M.marinum* strains (ATCC 927 and ATCC BAA-535/M) were within 0–1 dilution of the MICs of the 44 tested isolates and within the expected range.

The MICs at which 90 % of isolates were inhibited (MIC_90_) of amikacin (4 μg/mL), clarithromycin (2 μg/mL) and rifabutin (0.12 μg/mL) were below the CSLI breakpoint, whereas the MIC_90_ of ciprofloxacin (2 μg/mL), doxycycline (4 μg/mL), ethambutol (4 μg/mL), moxifloxacin (1 μg/mL), rifampin (1 μg/mL) and sulfamethoxazole (19 μg/mL) were close to the breakpoint [16]. The MIC_90_ of rifampin, rifabutin, moxifloxacin and trimethoprim (between 0.1 and 1 μg/mL) were lower than those of the other tested antibiotics.

The relatively high MIC_90_ of ethionamide (10 μg/mL), isoniazid (8 μg/mL) and streptomycin (16 μg/mL), for which breakpoints are not available, suggests that they are not good candidates for the treatment of *M. marinum* infection. In contrast, linezolid (MIC_90_: 2 μg/mL) was very effective against the different *M. marinum* isolates with MIC values among the lowest in our study.

The SLOMYCO Sensititre^®^ panel method (tested method) produced remarkably consistent and reproducible results (Table 3). Reproducibility with this method was 100 % in the case of ethionamide, isoniazid, moxifloxacin, rifampin, trimethoprim and sulfamethoxazole, and between 98 and 87 % for amikacin, ciprofloxacin, clarithromycin, doxycycline, ethambutol, linezolid, rifabutin and streptomycin (Table 3). The percentage essential agreement (±1 log_2_ dilution) between the MICs obtained with the tested and the reference methods (Table 4) greatly varied depending on the drug: from 98 % for ciprofloxacin and linezolid to 2.2 % for ethionamide. Good agreement percentages were obtained for moxifloxacin (91.3 %), isoniazid (87 %) and clarithromycin (85 %); whereas, agreement was lower for amikacin (76.1 %), rifampin (76.1 %), rifampin (76 %), trimethoprim (74 %), doxycycline (72 %), sulfamethoxazole (70 %), streptomycin (59 %) and particularly ethambutol (33 %). In the case of ethambutol and ethionamide, the SLOMYCO Sensititre^®^ panel method underestimated the MICs by 2–3 dilutions compared with the reference method. When the agar dilution and SLOMYCO Sensititre^®^ panel results were converted into interpretive categories (resistance/susceptibility) using the CLSI breakpoints, the category agreement was 100 % for amikacin, ciprofloxacin, clarithromycin, moxifloxacin, rifabutin and sulfamethoxazole–trimethoprim (SMT-TMP), 98 % for ethambutol and 96 % for rifampin (Table 5). Two very major discrepancies were observed for rifampin (2/46) and one for ethambutol (1/46). No agreement was observed between the two methods for doxycycline, with 46 (100 %) major discrepancies. The SLOMYCO Sensititre^®^ method overestimated the doxycycline MIC by 1 dilution; however, with this method, doxycycline MIC_90_ (8 μg/mL) was close to the susceptibility breakpoint obtained by using the agar dilution method (4 μg/mL).

**Table 3:** Reproducibility of the results (i.e., MIC, expressed in µg/mL, of the tested antibiotics in the 46 *M. marinum* isolates) obtained with the SLOMYCO Sensititre^®^ panel method

Antimicrobial agents	No. of results within log_2_ concentration difference of							% agreement (confidence interval)
	>−2	−2	−1	0	1	2	>2	
Amikacin	0	0	0	45	0	1	0	98 (87–100)
Ciprofloxacin	0	1	4	33	7	1	0	96 (84.3–99.3)
Clarithromycin	0	0	10	23	10	3	0	93.5 (81.1–98.3)
Doxycycline	0	0	6	38	1	1	0	97 (87–100)
Ethambutol	0	1	1	43	0	1	0	95.6 (84–99.2)
Ethionamide	0	0	4	29	13	0	0	100 (90.4–100)
Isoniazid	0	0	7	19	20	0	0	100 (86–99.3)
Linezolid	0	1	12	20	12	1	0	96 (87–100)
Moxifloxacin	0	0	7	36	3	0	0	100 (90.4–100)
Rifabutin	0	0	3	28	14	1	0	98 (87–100)
Rifampin	0	0	8	34	4	0	0	100 (90.4–100)
Streptomycin	0	1	5	29	11	0	0	98 (87–100)
Sulfamethoxazole	0	0	5	30	11	0	0	100 (90.4–100)
Trimethoprim	0	0	7	28	11	0	0	100 (90.4–100)

**Table 4:** Comparison of the MIC values obtained by using the SLOMYCO Sensititre^®^ panel and the agar dilution methods for 46 *M. marinum* isolates

Antimicrobial agents	No. of results within log_2_ concentration difference of							% essential agreement (confidence interval)
	>−2	−2	−1	0	1	2	>2	
Amikacin	0	11	34	1	0	0	0	76.1 (70–87)
Ciprofloxacin	0	1	32	11	2	0	0	98 (87–100)
Clarithromycin	4	3	23	15	1	0	0	85 (70.5–93.2)
Doxycycline	0	0	0	0	33	12	1	72 (56.3–83.5)
Ethambutol	12	19	14	1	0	0	0	33 (20–48.1)
Ethionamide	36	9	1	0	0	0	0	2.2 (0.1–13)
Isoniazid	0	6	27	11	2	0	0	87 (73–95)
Linezolid	0	0	10	20	15	1	0	98 (87–100)
Moxifloxacin	0	4	20	18	3	0	1	91.3 (87–100)
Rifabutin	0	0	0	0	35	11	0	76.1 (61–87)
Rifampin	0	11	20	15	0	0	0	76.1 (61–87)
Streptomycin	2	17	22	5	0	0	0	59 (43.3–72.7)
Sulfamethoxazole	2	10	15	13	4	1	1	70 (54.1–82)
Trimethoprim	3	9	26	6	2	0	0	74 (59–85.2)

**Table 5:** Comparison of the susceptibility testing results (resistance/susceptibility) and category errors (very major or major error) by using the SLOMYCO Sensititre^®^ panel (SSPM) and the agar dilution method (ADM) for 46 *M.* *marinum* isolates following the CLSI guidelines

Antimicrobial agents [breakpoints (µg/mL)]	% resistant ADM	% resistant SSPM	Category errors (N°)	% agreement
Amikacin (32)	0	0	None	100
Ciprofloxacin (2)	0	0	None	100
Clarithromycin (16)	0	0	None	100
Doxycycline (4)	0	100	Major (46)	0
Ethambutol (4)	2.2	0	Very major (1)	98
Moxifloxacin (2)	0	0	None	100
Rifabutin (2)	0	0	None	100
Rifampin (1)	4.3	0	Very major (2)	96
SMT-TMP (2/38)	0	0	None	100

*SMT–TM*P sulfamethoxazole–trimethoprim

## Discussion

The manufacturer’s guidelines (TREK Diagnostic Systems, Cleveland, OH) recommend the SLOMYCO Sensititre® method for antibiotic susceptibility testing in *M. marinum*, but studies evaluating the concordance with the reference agar dilution method have not been published yet. As the SLOMYCO Sensititre® panel technique presents several advantages (commercial availability; standardization; easy to set up and to interpret; and amenable to automation [2, 19] compared to the time-consuming and cumbersome agar dilution method, our objective was to compare the performance of these two methods.

The lack of difference between the MICs for the *M.marinum* isolates from infected humans and fish could be explained by the fact that most of the clinical samples were from patients who handled infected fish (from aquarium tanks or fish-related work) and, therefore, the antibiotic susceptibility profiles of the human isolates reflected those of the fish isolates.

Our results show that the reproducibility of the SLOMYCO Sensititre® results for the reference strains and the *M. marinum* isolates (from human and fish) was very good. All results of the independent tests were within the ±1 log_2_ dilution acceptable level of variation. Moreover, the level of agreement (±1 log_2_ dilution) between the results (MICs and interpretive categories) obtained with the SLOMYCO Sensititre® panel and the agar dilution methods was good for most of the antibiotics recommended by CLSI and ATS [12] for the treatment of (rifampin, rifabutin, amikacin, clarithromycin and sulfamethoxazole–trimethoprim) or with therapeutic potential (linezolid, isoniazid) for *M. marinum* infections. It should be noted that two very major errors were observed for rifampin in isolates in which the MIC for rifampin was just above the breakpoint (2 μg/mL) and that still belong to the wild type population [2]. The clinical significance of these data is unclear. Moreover, the MIC of the only rifampin-resistant *M.marinum* reported until now [14] was clearly above the breakpoint (>16 µg/mL). Unfortunately, we could not evaluate the ability of the SLOMYCO Sensititre® panel to detect resistant strains, because none of the isolates tested in this study was considered as antibiotic-resistant.

Conversely, poor agreement was observed for ethambutol. The MICs for ethambutol obtained with the SLOMYCO Sensititre® panel were 2–3 dilutions lower than those obtained with the reference method. However, this poor agreement resulted in only one very major error, when MICs were converted into interpretive categories. This discrepancy corresponds to a strain with a MIC just above the breakpoint (16 µg/mL) (Table 1).

In the case of doxycycline, no agreement was observed between methods resulting in 46 major errors. This could be explained by (i) the overestimation of doxycycline MICs by the SLOMYCO Sensititre® panel that could be due to the medium or pH, and (ii) the fact that the MIC_50_ and MIC_90_ were similar to the breakpoint value. Among the antibiotics recommended by CLSI and on the basis of in vitro susceptibility testing, our results confirm that rifampin, rifabutin, amikacin, clarithromycin and sulfamethoxazole–trimethoprim are good options for the treatment of *M. marinum* infections, as reported in previous studies [2, 4, 11, 14, 15, 19]. Sulfamethoxazole and trimethoprim showed good in vitro activity against *M. marinum* and could be considered as an alternative treatment [19]. The present study brings new data on *M. marinum* susceptibility pattern to ethionamide, streptomycin and linezolid. The American Thoracic Society recommendations for the treatment of some nontuberculous mycobacteria (NTM) infections include the use of streptomycin (for rifampin-resistant *M. kansassii* infection) and ethionamide (for *M. malmoense* infection) [7, 12]. However, the lack of clinical experience in *M. marinum* infections and the absence of breakpoints for NTM susceptibility and resistance to these two antibiotics did not allow predicting their potential efficiency in *M. marinum* infections [16]. Linezolid has been reported to be effective against mycobacteria (*M. chelonae* and *M. marinum*) [6, 7] and for treating skin and soft tissue infections [6, 7]. In our study, linezolid was one of the most active antimicrobial agents in agreement with the low MIC determined in a previous study [4]. Despite the lack of breakpoint values for *M. marinum*, linezolid may be an interesting alternative therapeutic agent due to its pharmacological properties. This study confirmed that *M. marinum* is resistant to isoniazid and ethambutol [7] and the observed *M. marinum* susceptibility pattern corresponded to the wild type phenotype, as previously reported [2, 7, 14, 18, 19]. As the ethambutol MIC_90_ was close to the breakpoint and clear evidence on the clinical efficacy of this antibiotic are lacking, it should not be recommended as a first-line drug for *M.marinum* infection treatment.

## Conclusion

In this study, we evaluated the SLOMYCO Sensititre^®^ panel method for susceptibility testing in 44 *M.marinum* isolates (from humans and fish) relative to the reference agar dilution method. Our results indicate that the SLOMYCO Sensititre^®^ panel method could provide a potential alternative to the reference agar dilution method, when DST in *M.marinum* is required, except for doxycycline. If doxycycline susceptibility needs to be tested, the use of another method (broth microdilution) is more appropriate.

## Consent

Written informed consent was obtained from the patients for the publication of this report.
